# 6PPD-quinone exposure and Alzheimer’s disease: insights from integrative network pharmacology, transcriptomics, machine learning, and molecular docking

**DOI:** 10.1515/med-2026-1477

**Published:** 2026-06-24

**Authors:** Chun Zhang, Jingqi Zhang

**Affiliations:** Chongqing Three Gorges Medical College, Chongqing, China; School of Clinical Medicine, Chengdu University of Traditional Chinese Medicine, Chengdu, China; Department of Otorhinolaryngology, Hospital of Chengdu University of Traditional Chinese Medicine, Chengdu, China

**Keywords:** 6PPD-quinone, Alzheimer’s disease, network pharmacology, neuroinflammation, oxidative stress

## Abstract

**Objectives:**

To systematically investigate the molecular associations between 6PPD-quinone (6PPD-Q), an environmental transformation product of the tire antioxidant 6PPD, and Alzheimer’s disease (AD) pathogenesis.

**Methods:**

An integrative strategy combining network pharmacology, transcriptomic validation, and machine learning was employed. Intersecting targets were identified through multi-database mining, followed by functional enrichment and protein–protein interaction (PPI) network analyses. Transcriptomic validation, SHAP-based XGBoost analysis, Mendelian randomization, and molecular docking were performed to evaluate target expression, diagnostic value, causal associations, and binding affinities.

**Results:**

A total of 92 intersecting targets were identified, enriched in synaptic structures, kinase activity, neuroinflammation, and apoptotic pathways. PPI analysis revealed 23 core targets, with *NFKB1*, *GSK3B*, and *PIK3CA* as key hub genes enriched in the cerebral cortex and basal ganglia. Transcriptomic data confirmed differential expression of core targets in AD. SHAP analysis identified *PTGS2*, *KIT*, *PIK3CA*, *NFE2L2*, and *NFKB1* as high-value diagnostic predictors. Mendelian randomization supported a causal association between *NFKB1* brain expression and AD risk. Molecular docking confirmed strong binding of 6PPD-Q to *PTGS2*, *GSK3B*, and *NFE2L2*.

**Conclusions:**

This study provides the first systematic characterization of the molecular mechanisms by which 6PPD-Q may contribute to AD pathogenesis, potentially through inducing oxidative stress, activating neuroinflammation, and disrupting kinase signaling networks.

## Introduction

Alzheimer’s disease (AD) is the most prevalent neurodegenerative disorder worldwide, characterized by progressive cognitive decline, memory impairment, and behavioral abnormalities [1]. According to the World Health Organization, approximately 55 million people currently suffer from dementia globally, with AD accounting for 60–70 % of cases, and this number is projected to reach 139 million by 2050 [2]. The pathological hallmarks of AD include extracellular amyloid-β (Aβ) plaques, intracellular neurofibrillary tangles composed of hyperphosphorylated tau protein, synaptic dysfunction, and neuroinflammation [3]. Despite decades of research, the etiology of AD remains incompletely understood, and current therapeutic interventions can only alleviate symptoms without halting disease progression [4]. While genetic factors contribute significantly to AD risk, accumulating evidence suggests that environmental exposures may play an underappreciated role in AD pathogenesis [5]. Epidemiological studies have linked air pollution, heavy metals, and pesticides to increased AD incidence, highlighting the importance of identifying novel environmental risk factors [6]. In particular, emerging environmental pollutants derived from anthropogenic activities are receiving increasing attention due to their potential neurotoxic effects.

N-(1,3-dimethylbutyl)-N′-phenyl-p-phenylenediamine quinone (6PPD-Q) is a transformation product of 6PPD, an antiozonant widely used in tire rubber manufacturing [7]. Since the discovery that 6PPD-Q can cause acute mortality in coho salmon at environmentally relevant concentrations, this compound has emerged as a pollutant of significant ecological concern [8]. 6PPD-Q is released into the environment through tire wear particles and stormwater runoff, resulting in widespread contamination of both aquatic and terrestrial ecosystems. Recent studies have detected 6PPD-Q in various environmental matrices including road dust, surface water, and sediments, as well as in human biological samples, indicating that humans may be exposed to this substance through multiple pathways [9].

The structural features of 6PPD-Q, particularly its quinone moiety, suggest a potential to induce oxidative stress and protein modification [10]. Quinone-containing compounds are known to undergo redox cycling, generate reactive oxygen species (ROS), and form covalent adducts with cellular proteins [11]. These mechanisms are particularly relevant to neurodegenerative diseases, as the brain is highly susceptible to oxidative damage due to its high metabolic rate, abundant polyunsaturated fatty acids, and relatively limited antioxidant defenses [12]. Furthermore, studies have shown that 6PPD-Q can penetrate biological membranes and accumulate in lipid-rich tissues [13]. Notably, it has been demonstrated to cross the blood-brain barrier in mice within 0.5 h of exposure, raising concerns about its potential to reach the central nervous system [14].

Despite the increasing environmental prevalence of 6PPD-Q and its documented toxicity to aquatic species, the potential effects of this emerging pollutant on human neurological health, particularly its relationship with AD, remain largely unexplored. Given the shared pathophysiological mechanisms between environmental neurotoxicant exposure and AD including oxidative stress, neuroinflammation, and disruption of protein homeostasis investigating the molecular links between 6PPD-Q and AD carries significant public health implications.

Network pharmacology represents a powerful systems biology approach for elucidating the complex interactions between chemicals and biological systems [15]. By integrating target prediction, protein–protein interaction analysis, and pathway enrichment, this approach enables comprehensive identification of the molecular mechanisms underlying chemical-disease associations [16]. When combined with transcriptomic validation and Mendelian randomization analysis, network pharmacology can provide robust evidence for potential causal relationships between molecular perturbations and disease outcomes [17].

**Figure 1:**
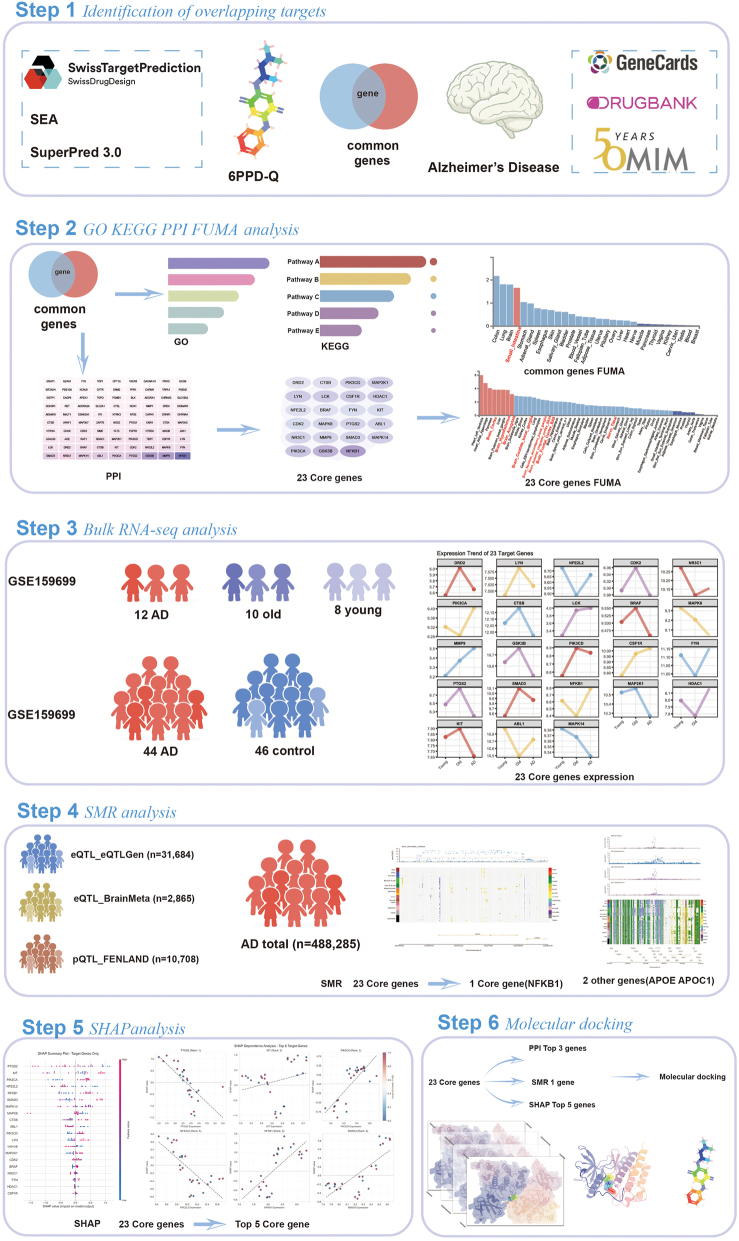
Schematic workflow of the integrated bioinformatics analysis investigating the molecular relationship between 6PPD-Q exposure and Alzheimer’s disease.

This study employed an integrative network pharmacology approach to systematically investigate the potential molecular mechanisms linking 6PPD-Q exposure to AD pathogenesis. We identified common targets between 6PPD-Q and AD through comprehensive database mining, constructed protein–protein interaction (PPI) networks to screen for core hub genes, and performed functional enrichment analysis to characterize the relevant biological pathways. The expression patterns of identified core targets were validated using independent transcriptomic datasets and protein expression data. Machine learning-based SHAP analysis was applied to evaluate the diagnostic predictive value of core genes. Additionally, summary-data-based Mendelian randomization (SMR) analysis was conducted to explore potential causal relationships between gene expression and AD risk. Finally, molecular docking simulations were performed to assess direct binding interactions between 6PPD-Q and key target proteins. This study aims to provide the first systematic characterization of the molecular mechanisms through which 6PPD-Q, an emerging environmental pollutant, may participate in AD-related pathological processes. Our findings may offer new insights into environmental risk factors for neurodegenerative diseases and identify potential therapeutic targets for intervention strategies. The research process of this study is shown in Figure 1.

## Materials and methods

### Acquisition of 6PPD-Q-related targets

The potential targets of 6PPD-Q were obtained through an integrated strategy using three complementary databases and prediction tools. The SMILES structure of polyethylene terephthalate was retrieved from PubChem (https://pubchem.ncbi.nlm.nih.gov/). SwissTargetPrediction (http://www.swisstargetprediction.ch/) [18] was used to predict potential human targets by inputting the SMILES structure, with a probability threshold set at >0. Similarity ensemble approach (SEA, http://sea.bkslab.org/) [19] was employed for ligand-based target prediction with a p-value threshold <0.05. SuperPred 3.0 (https://prediction.charite.de/subpages/target_prediction.php) [20] was additionally utilized for target prediction based on structural similarity, with targets showing probability >0 being retained.

### Acquisition of Alzheimer’s disease-related genes

To construct a comprehensive library of genes related to AD, data were cross-referenced from three authoritative repositories: GeneCards, DrugBank, and OMIM. Specifically, candidates were retrieved from the GeneCards database (https://www.genecards.org/) [21] using “Alzheimer’s disease” as the primary search term. To ensure data quality and relevance, only genes with a relevance score >15 were retained. Additionally, disease-related targets were supplemented by querying the DrugBank (https://go.drugbank.com/) [22] and OMIM (https://www.omim.org/) [23] databases to capture clinically validated and genetically linked targets.

### Identification of overlapping targets and functional enrichment analysis

To identify the shared therapeutic targets, an intersection analysis was conducted among 6PPD-Q-related targets and AD-associated genes using the InteractiVenn online platform (https://www.interactivenn.net/) [24]. The overlapping gene set was subsequently subjected to systematic functional annotation and pathway enrichment analyses.

GO and KEGG enrichment analyses were conducted with the clusterProfiler package [25]. Gene symbols were converted to Entrez IDs using the bitr function with the org.Hs.eg.db annotation database. GO terms were classified into three categories: biological process (BP), cellular component (CC), and molecular function (MF), while KEGG analysis focused on identifying disease-relevant signaling pathways. Enrichment significance was assessed with thresholds of adjusted p-value <0.05 and q-value <0.2, with multiple testing correction performed using the Benjamini-Hochberg (BH) method. Results were visualized using the ggplot2 package.

### Protein–protein interaction network construction and core target screening

The overlapping targets were subjected to protein–protein interaction (PPI) network analysis using the STRING database (https://string-db.org/) [26] with the species restricted to *Homo sapiens*. The interaction data were imported into Cytoscape (version 3.9.1) [27] for network visualization and topological analysis.

Hub genes were identified based on degree centrality calculated by the CytoNCA plugin [27]. A stepwise screening approach was applied: nodes ranking in the top 50 % by degree value were first selected, followed by a second filtering round retaining the top 50 % of the remaining nodes [28]. This two-step strategy is mathematically equivalent to applying the median degree value as a threshold at each filtering stage, which is a widely adopted non-parametric approach in network pharmacology research. Given that degree distributions in PPI networks typically follow a power-law distribution, the median serves as a more robust threshold than the mean, as it is less susceptible to distortion by high-degree hub outliers, and avoids the arbitrariness of fixed numerical cut-offs that vary substantially across networks of different scales. The final gene set obtained through this dual-filtering process was considered as preliminary core targets for further investigation.

### Tissue expression enrichment analysis using FUMA

Tissue-specific expression patterns of candidate genes were evaluated using the FUMA platform (https://fuma.ctglab.nl/) [29]. Both the overlapping genes and the core hub targets were separately analyzed through the GENE2FUNC module. Expression enrichment was assessed across 54 tissue types and 8 major tissue categories using the GTEx v8 database (https://gtexportal.org/home/) [30], with MHC region genes excluded to reduce immunogenetic confounding. Statistical significance was determined using the Benjamini-Hochberg method with FDR<0.05. Special attention was given to enrichment patterns in brain and nervous system tissues to explore the neurological effects of 6PPD-Q related to AD.

### Transcriptomic validation of core target genes

To validate the expression of core targets in AD, two bulk RNA-seq datasets were obtained from the GEO database (https://www.ncbi.nlm.nih.gov/geo/).

**GSE159699** [31] included postmortem lateral temporal lobe samples from 12 AD patients, 10 elderly controls, and 8 young controls, sequenced on Illumina NextSeq 500. All 23 core genes were analyzed. Raw counts were normalized using DESeq2 [32] with variance stabilizing transformation (VST). Low-expressed genes (mean counts <10 in fewer than 3 samples) were filtered. PCA was performed to evaluate sample clustering. Differential expression analysis was conducted across three groups (young vs. old vs. AD) with thresholds of adjusted p-value <0.05 and |log2FC|>0.5 (Benjamini-Hochberg correction). Spearman correlation analysis was used to construct a gene correlation matrix among the 23 targets.

**GSE174367** [33] contained 44 AD and 46 control samples. Of the 23 core genes, 20 were present in this dataset. Differential expression analysis comparing AD vs. controls was performed using the same pipeline to validate findings from GSE159699. Results were visualized using ggplot2, pheatmap, corrplot, and ggpubr packages in R.

### Protein expression validation using Human Protein Atlas

To investigate the protein expression patterns of key core targets in brain tissues, immunohistochemistry data were retrieved from the Human Protein Atlas (HPA) database (https://www.proteinatlas.org/) [34]. Based on network centrality analysis and differential expression results, the top two ranked core genes, *NFKB1* and *GSK3B*, were selected for protein-level validation. Brain tissue expression images were obtained from the HPA Tissue Atlas section, which provides immunohistochemical staining data from normal human tissues. Expression levels and cellular localization patterns in cerebral cortex and other brain regions were examined to validate the relevance of these targets in brain pathology.

### SHAP analysis and machine learning model construction

To systematically evaluate the predictive value of the 23 candidate genes for AD diagnosis, a machine learning-based Shapley additive explanations (SHAP) analysis was performed [35]. Expression profiles of the target genes were extracted from the GSE174367 dataset, yielding 20 available genes across 90 samples.

An XGBoost classifier was employed to construct the predictive model [36]. The dataset was randomly split into training (70 %, n=63) and test (30 %, n=27) sets using stratified sampling to maintain class proportions. Model hyperparameters were set as follows: n_estimators=100, max_depth=3, and learning_rate=0.1. SHAP values were calculated using the TreeExplainer algorithm to quantify the contribution of each gene to model predictions. Positive SHAP values indicated that gene expression promoted AD classification, while negative values suggested promotion of control classification. Feature importance was assessed by calculating the mean absolute SHAP value for each gene. Additionally, independent samples *t*-test was applied to evaluate differential expression between AD and control groups (significance level alpha=0.05). Model performance was evaluated using accuracy and area under the receiver operating characteristic curve (AUC). All analyses were conducted in Python 3.8 using shap, xgboost, and scikit-learn packages.

### Summary-data-based Mendelian randomization (SMR) analysis

To investigate potential causal relationships between gene expression/protein levels and AD risk, SMR analysis was performed using the SMR online platform (https://yanglab.westlake.edu.cn/software/smr) [17]. AD GWAS summary statistics were obtained from the IEU OpenGWAS database (ID: ieu-b-5067) [37] as the outcome, including 488,285 European individuals (954 AD cases and 487,331 controls). Three molecular QTL datasets were employed as exposures: (1) eQTL_eQTLGen (n=31,684), comprising whole blood eQTL data from the eQTLGen Consortium covering approximately 88 % of genes across 37 European cohorts [38]; (2) eQTL_BrainMeta (n=2,865), containing brain cortex eQTL data from 2,865 RNA-seq samples of 2,443 unrelated European individuals with 16,704 identified eGenes [39]; and (3) pQTL_FENLAND (n=10,708), including plasma protein QTL data from the Fenland study measuring approximately 4,775 proteins using the Olink platform [40]. The minimum allele frequency (MAF) threshold was set at 0.01, with an allele frequency difference threshold of 0.99. P_SMR<0.05 was considered statistically significant for causal associations. The heterogeneity in dependent instruments (HEIDI) test was applied to distinguish pleiotropy from linkage disequilibrium, where P_HEIDI>0.05 indicated that the observed association was not driven by linkage disequilibrium, supporting a potential causal relationship between gene/protein expression and AD rather than horizontal pleiotropy.

### Molecular docking

To evaluate the binding interactions between 6PPD-Q and core target proteins, molecular docking analysis was conducted. The SMILES structure of 6PPD-Q was retrieved from the PubChem database (https://pubchem.ncbi.nlm.nih.gov/). Three-dimensional structures of target proteins were obtained from the UniProt database (https://www.uniprot.org/) and RCSB PDB database (https://www.rcsb.org/). Protein structures were preprocessed using PyMOL (v2.5) to remove water molecules and co-crystallized ligands. The 3D conformation of 6PPD-Q was generated and energy-minimized using the MM2 force field in Chem3D. Semi-flexible docking was performed using AutoDock Vina (v1.2.5) with default grid spacing (0.375 Å). Binding affinity (kcal/mol) was used to rank docking conformations, with lower values indicating stronger binding capacity. Docking results were visualized using PyMOL, and protein-ligand interactions including hydrogen bonds, hydrophobic contacts, and pi-pi stacking were analyzed using the PLIP online platform (https://plip-tool.biotec.tu-dresden.de/) [41].

### *In silico* gene perturbation analysis in single-nucleus transcriptomic data

To assess the potential functional impact of the identified key targets within the central nervous system, we performed *in silico* gene perturbation analysis using a single-nucleus RNA-sequencing (snRNA-seq) dataset of the human prefrontal cortex (GSE157827), comprising 163,824 nuclei from 12 Alzheimer’s disease (AD) patients and 9 age-matched normal controls. Raw UMI count matrices were normalized to 10,000 counts per nucleus followed by log1p transformation. The top 3,000 highly variable genes were identified using the Seurat v3 variance-stabilizing method. To mitigate inter-individual batch effects, principal component analysis (50 components) was performed on highly variable genes, and the resulting embeddings were corrected using Harmony with sample identity as the batch covariate. UMAP embedding was subsequently computed on the Harmony-corrected representation. Cell type annotation was performed by scoring each Leiden cluster against canonical marker gene sets for seven major brain cell types: excitatory neurons (*SLC17A7, SATB2, SNAP25, NEUROD6*), inhibitory neurons (*GAD1, GAD2, DLX2, LAMP5*), astrocytes (*GFAP, AQP4, SLC1A3, SLC1A2*), microglia (*C1QA, C1QB, TREM2, P2RY12, CSF1R*), oligodendrocytes (*MOG, MBP, PLP1, CLDN11*), oligodendrocyte precursor cells (*PDGFRA, CSPG4, OLIG1, OLIG2*), and endothelial cells (*CLDN5, PECAM1, FLT1, VWF*). Each cluster was assigned the cell type identity corresponding to the highest mean gene-set enrichment score.

For *in silico* knockout (KO) simulation, we focused on microglia – the primary immune effector cells of the CNS and central mediators of neuroinflammation in AD. Microglial nuclei (n=7,282) were randomly subsampled to 2,000 cells for computational efficiency (random seed=42). For each key gene (*NFKB1*, *GSK3B*, *PIK3CA*, *NFE2L2*, *PTGS2*, and *KIT*), a co-expression network was constructed by computing pairwise Pearson correlation coefficients between the key gene and all highly variable genes across microglial nuclei. The top 50 genes ranked by absolute correlation coefficient were retained as putative downstream regulatory targets. The predicted transcriptional effect of gene knockout was estimated under a simplified linear perturbation model: ΔE_target=−r × E_target, where r denotes the Pearson correlation coefficient between the KO gene and the target gene, and E_target represents the mean log-normalized expression of the target gene in the respective condition. Under this model, a positive ΔE indicates predicted de-repression upon KO, whereas a negative ΔE indicates predicted transcriptional suppression. It should be noted that this framework assumes linearity and does not capture non-linear regulatory interactions or indirect network effects; results should therefore be interpreted as hypothesis-generating rather than mechanistically definitive. Predicted KO effects were computed separately for AD and control nuclei to identify disease-specific perturbation outcomes.

## Outcome

### Identification of common targets between 6PPD-Q and Alzheimer’s disease and GO/KEGG enrichment analysis

**Figure 2:**
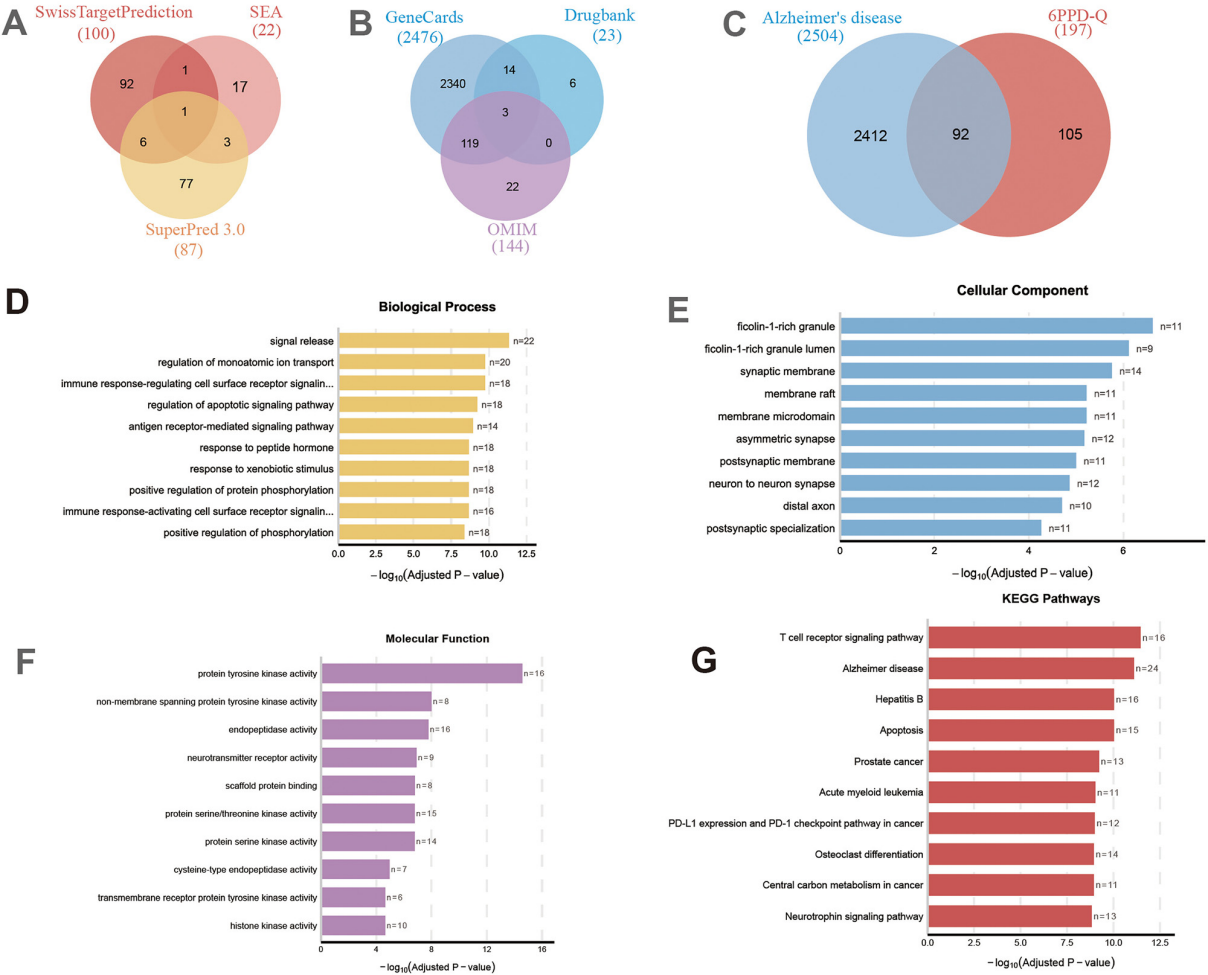
Identification of common targets between 6PPD-Q and Alzheimer’s disease and GO/KEGG enrichment analysis. (A) Predicted targets of 6PPD-Q. (B) AD-related genes retrieved from disease databases. (C) Venn diagram illustrating overlapping targets between 6PPD-Q and AD. (D–F) GO enrichment analysis results for (D) biological process, (E) molecular function, and (F) cellular component categories. (G) KEGG pathway enrichment analysis of overlapping targets.

To identify potential molecular targets linking 6PPD-Q exposure to AD pathogenesis, we performed comprehensive target prediction and disease gene mining. For 6PPD-Q, SwissTargetPrediction, SEA, and SuperPred 3.0 predicted 100, 22, and 87 potential targets, respectively (Figure 2A; Supplementary Data 1). For AD-related genes, 2,476, 23, and 144 genes were retrieved from GeneCards, DrugBank, and OMIM databases, respectively (Figure 2B; Supplementary Data 2). Intersection analysis identified 92 overlapping targets between 6PPD-Q and AD (Figure 2C).

GO enrichment analysis revealed that these common targets were significantly enriched in biological processes related to immune response regulation, apoptotic signaling pathway regulation, and positive regulation of protein phosphorylation (Figure 2D). Cellular component analysis showed predominant enrichment in synaptic structures, including synaptic membrane, postsynaptic membrane, asymmetric synapse, and distal axon (Figure 2E). Molecular function analysis highlighted protein tyrosine kinase activity, protein serine/threonine kinase activity, and neurotransmitter receptor activity as the most significantly enriched terms (Figure 2F).

KEGG pathway enrichment analysis identified Alzheimer’s disease pathway as the most significantly enriched (n=24 genes), followed by T cell receptor signaling pathway, apoptosis, and neurotrophin signaling pathway (Figure 2G; Supplementary Data 3). These results suggest that 6PPD-Q may influence AD-related pathological processes through mechanisms involving neuroinflammation, synaptic dysfunction, abnormal kinase-mediated phosphorylation, and apoptotic signaling.

### PPI network topology analysis and tissue-specific expression enrichment

**Figure 3:**
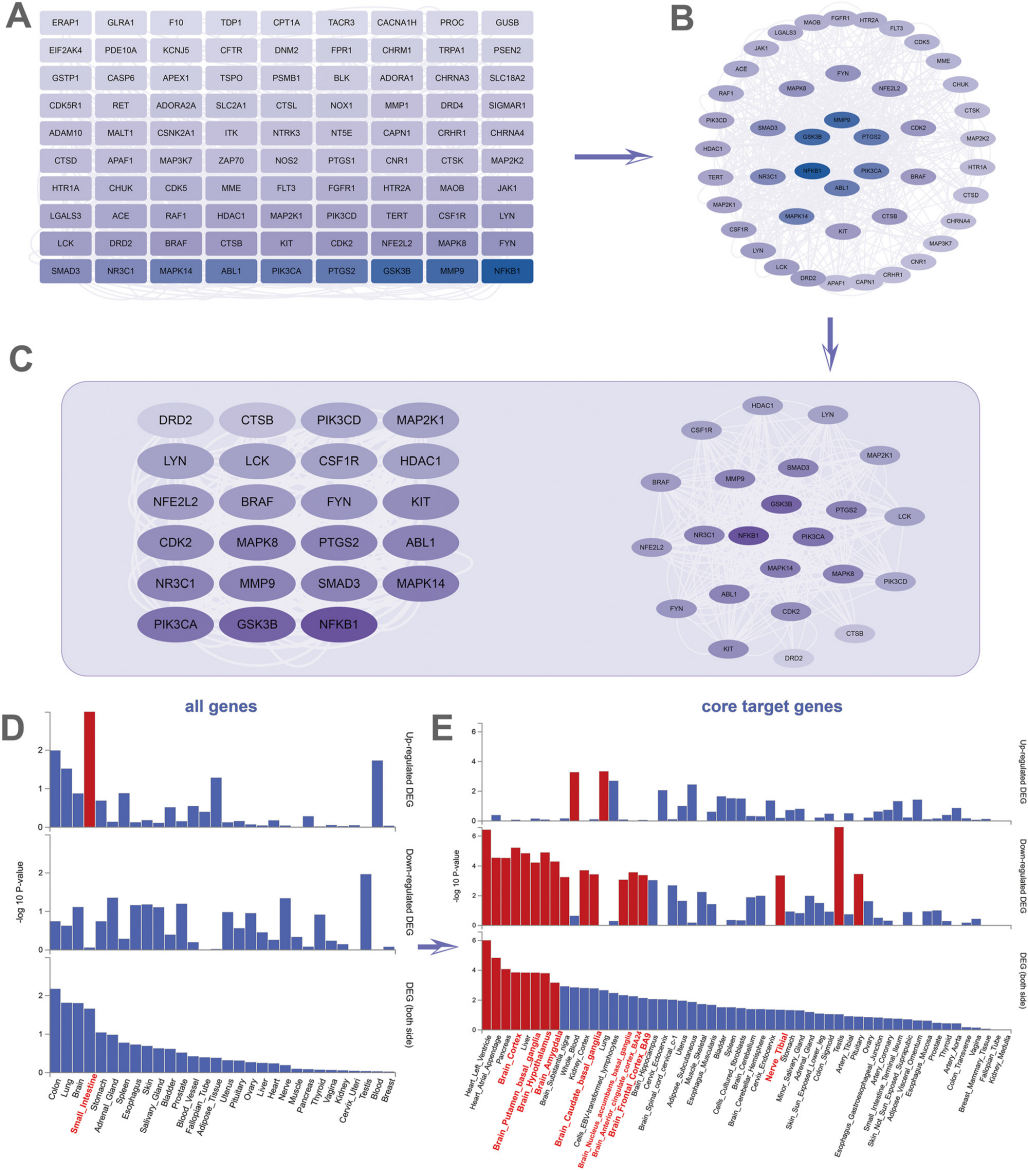
PPI network topology analysis and tissue-specific expression enrichment of common targets. (A–C) Progressive filtering of the PPI network based on degree centrality: (A) All 92 overlapping targets, (B) top 50 % nodes after first-round filtering, and (C) top 25 % core targets after second-round filtering. (D–E) FUMA tissue expression enrichment analysis for (D) all 92 overlapping targets and (E) 23 core hub targets across 54 tissue types.

To identify core targets and explore their regulatory networks, we constructed a PPI network using the 92 overlapping targets. The initial network comprised all target interactions (Figure 3A), which was then progressively filtered based on degree centrality. The first-round filtering retained the top 50 % nodes (Figure 3B), followed by a second-round screening to obtain the top 25 % nodes, yielding 23 core targets (Figure 3C; Supplementary Data 4).

Topological analysis using the CytoNCA plugin revealed that *NFKB1*, *GSK3B*, and *PIK3CA* ranked as the top three hub genes based on multiple centrality metrics. *NFKB1* exhibited the highest degree centrality (Degree=40) and closeness centrality (CC=0.917), followed by *GSK3B* (Degree=36, CC=0.846) and *PIK3CA* (Degree=32, CC=0.786). All three hub genes demonstrated high clustering coefficients (0.516–0.600) and low eccentricity (2), indicating their central positions in the network and potential key regulatory roles. Other notable hub genes included *MAPK14* (Degree=30), *NR3C1, MMP9, ABL1, MAPK8, PTGS2,* and *SMAD3* (all with Degree=28).

FUMA tissue expression enrichment analysis revealed distinct expression patterns between all overlapping targets and core targets. The 92 overlapping targets showed highest expression enrichment in small intestine (Figure 3D). In contrast, the 23 core targets demonstrated significant enrichment specifically in brain-related tissues (Figure 3E), including brain cortex, brain putamen (basal ganglia), brain hypothalamus, brain amygdala, brain caudate (basal ganglia), brain nucleus accumbens (basal ganglia), brain anterior cingulate cortex, and brain frontal cortex, as well as peripheral nerve tissue (tibial nerve). This brain-specific enrichment pattern strongly supports the neurological relevance of these core targets in AD pathogenesis and suggests that 6PPD-Q may exert neurotoxic effects through dysregulation of these key genes in AD-vulnerable brain regions.

### Transcriptomic and protein expression validation of core target genes

To validate the expression patterns of the 23 core targets in AD, we performed transcriptomic analysis using two independent GEO datasets and protein-level validation using the Human Protein Atlas.

**Figure 4:**
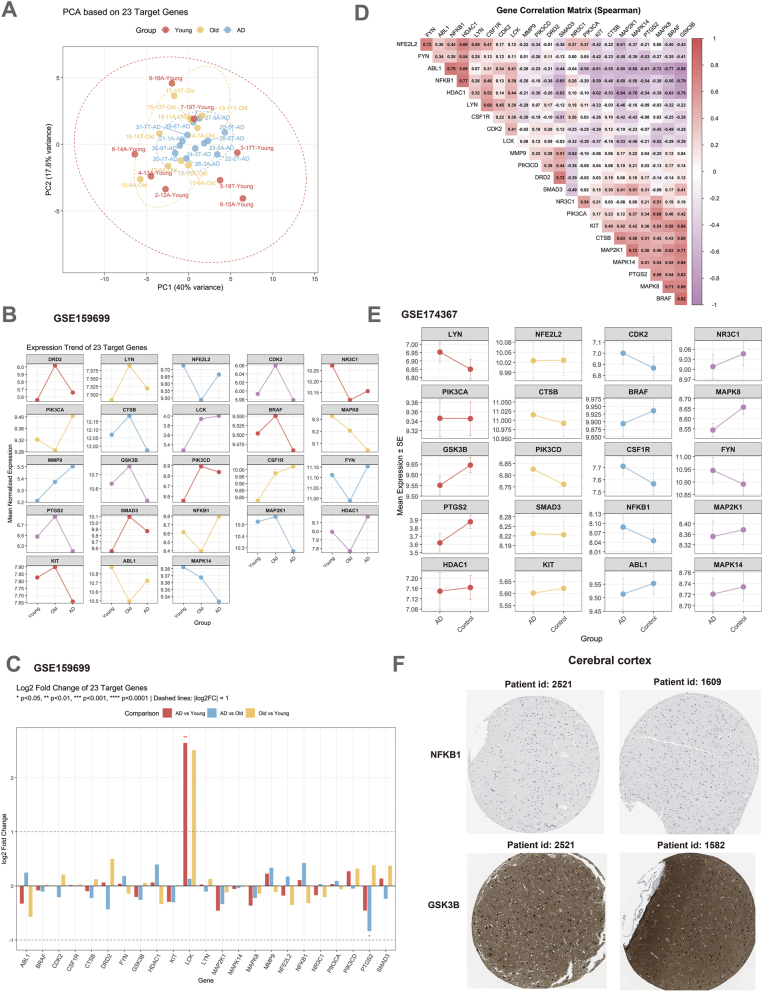
Transcriptomic and protein-level validation of core target genes in Alzheimer’s disease. (A) PCA plot of GSE159699 samples grouped by disease status. (B) Expression profiles of core hub genes across groups in GSE159699. (C) Differential expression analysis results in GSE159699. (D) Spearman correlation matrix of the 23 core target genes. (E) Validation of differential expression patterns in the independent GSE174367 dataset. (F) Immunohistochemical staining of *NFKB1* and *GSK3B* in human brain tissues from the Human Protein Atlas database.

Principal component analysis (PCA) of GSE159699 demonstrated that PC1 and PC2 explained 40 and 17.9 % of the variance, respectively (Figure 4A). Expression profiling of key hub genes revealed distinct patterns across groups (Figure 4B). *GSK3B* and *KIT* showed decreased expression in AD, while *NFKB1* and *PIK3CA* exhibited highest expression in AD group. Differential expression analysis identified *PTGS2* and *LCK* as the most significantly altered genes (Figure 4C). *LCK* was significantly upregulated in AD vs. young (log2FC>1, p<0.01), while *PTGS2* was significantly downregulated in AD vs. old (p<0.05).

Spearman correlation analysis revealed strong co-expression relationships among core genes (Figure 4D). *GSK3B* showed strong positive correlations with *BRAF* (r=0.82), MAPK8 (r=0.68), and *PTGS2* (r=0.63), but strong negative correlation with *NFKB1* (r=−0.79). *PIK3CA* was positively correlated with *MAPK8* (r=0.54), and *NFKB1* showed strong positive correlation with *ABL1* (r=0.76).

Validation in GSE174367 confirmed the expression patterns observed in GSE159699 (Figure 4E). *PTGS2* was significantly elevated in AD group, *NFKB1* showed higher expression in AD, and *GSK3B* was decreased in AD, consistent with our previous findings.

Immunohistochemistry data from the Human Protein Atlas confirmed protein expression of top hub genes in brain tissues (Figure 4F). *NFKB1* was predominantly expressed in neuronal cells of the cerebral cortex. *GSK3B* exhibited high protein expression levels in the cerebral cortex, with strong cytoplasmic/membrane staining in nearly all neurons and neuropil regions, moderate expression in glial cells, and weak expression in endothelial cells. These protein expression patterns validate the neurological relevance of these core targets in AD pathology.

### Machine learning-based SHAP analysis for core gene diagnostic value

To systematically evaluate the diagnostic predictive value of the 23 core genes for AD, we performed SHAP analysis using an XGBoost classifier on the GSE174367 dataset (20 genes available).

**Figure 5:**
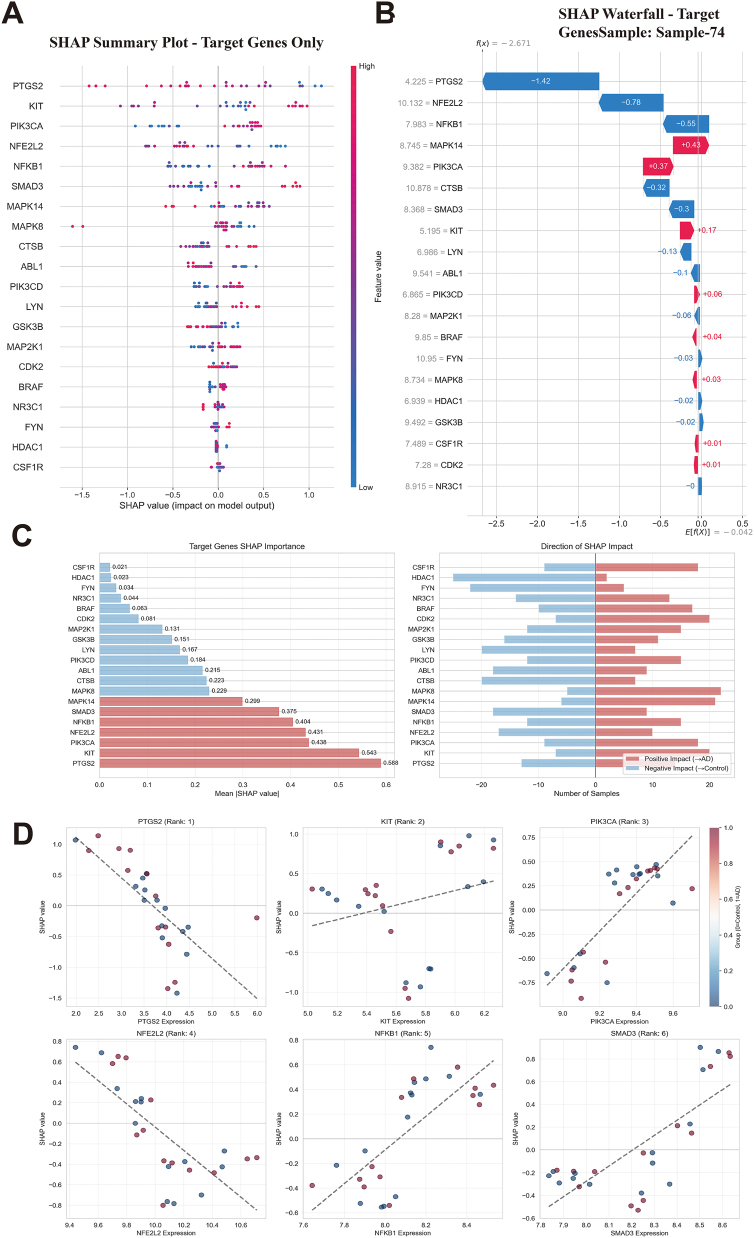
Machine learning-based SHAP analysis evaluating the diagnostic value of core target genes for Alzheimer’s disease. (A) SHAP summary plot illustrating the distribution and magnitude of SHAP values for all 20 available target genes, with color representing expression level. (B) SHAP waterfall plot for a representative control sample demonstrating individual gene contributions to the final prediction score. (C) Left: mean absolute SHAP value ranking reflecting overall feature importance. Right: directional impact distribution of each gene, with red indicating AD-promoting effects and blue indicating control-promoting effects. (D) SHAP dependence plots for the top six genes demonstrating the relationship between gene expression levels and corresponding SHAP values.

The SHAP summary plot revealed that *PTGS2*, *KIT*, *PIK3CA*, *NFE2L2*, and *NFKB1* were the top five contributors to model prediction (Figure 5A). Notably, *PTGS2* and *NFE2L2* showed a distinct pattern where high expression corresponded to negative SHAP values, pushing predictions toward “Control” classification. Conversely, high expression of *KIT*, *PIK3CA*, and *NFKB1* corresponded to positive SHAP values, driving predictions toward “AD” classification.

The waterfall plot for a representative control sample (Sample-74) illustrated individual gene contributions to the final prediction (Figure 5B). The final prediction value f(x)=−2.671, substantially below the baseline E[f(X)]=−0.042, indicated strong classification as “Control”. *PTGS2* (−1.42) and *NFE2L2* (−0.78) were the primary drivers, while *MAPK14* (+0.43) and *PIK3CA* (+0.37) provided positive contributions.

Feature importance ranking based on mean absolute SHAP values identified *PTGS2* (0.588), *KIT* (0.543), *PIK3CA* (0.438), *NFE2L2* (0.431), and *NFKB1* (0.404) as the most important predictive genes (Figure 5C, left). Directional impact analysis showed that *PTGS2* predominantly exhibited control-promoting effects, while *KIT*, *PIK3CA*, and *MAPK14* showed AD-promoting effects in the majority of samples (Figure 5C, right).

SHAP dependence plots demonstrated clear expression-SHAP relationships for the top six genes (Figure 5D). *PTGS2* and *NFE2L2* showed significant negative correlations, with high expression predicting “Control” status. In contrast, *KIT*, *PIK3CA*, *NFKB1*, and *SMAD3* exhibited significant positive correlations, with high expression predicting “AD” classification.

In summary, integrating PPI network topology with SHAP-based feature importance analysis identified *PTGS2*, *KIT*, *PIK3CA*, *NFE2L2*, and *NFKB1* as the five key genes with the highest predictive value for AD classification, representing potential core targets linking 6PPD-Q exposure to AD pathogenesis.

### Summary-data-based Mendelian randomization (SMR) analysis

To investigate potential causal relationships between core gene expression and AD risk, we performed SMR analysis integrating eQTL, pQTL, and AD GWAS data.

SMR analysis confirmed a potential causal association between *NFKB1* brain tissue eQTL and AD GWAS signals (p_SMR=0.032, p_HEIDI=0.513, nsnp_HEIDI=20) (Supplementary Data 5). The top SNP rs1005819 (MAF=0.422) served as a strong cis-eQTL for *NFKB1* (p_eQTL=2.39 × 10^−22^), with the T allele positively associated with *NFKB1* expression. The non-significant HEIDI test (p>0.05) suggests that the observed association is unlikely driven by linkage disequilibrium, supporting genetic evidence for *NFKB1* expression alterations as a causal factor in AD risk. This finding is consistent with the transcriptomic and SHAP analyses showing *NFKB1* dysregulation in AD.

**Figure 6:**
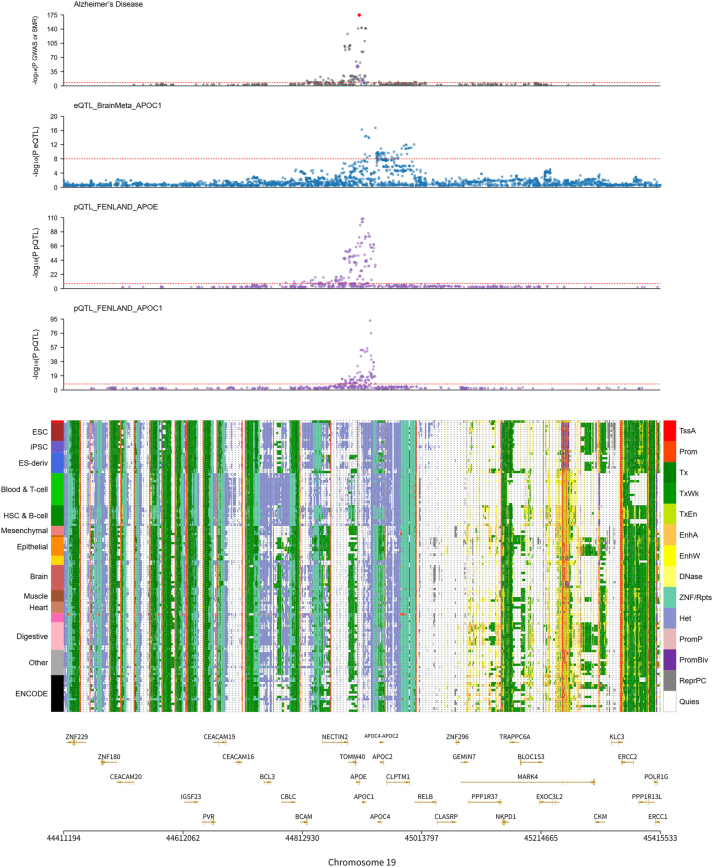
Summary-data-based Mendelian randomization analysis of the *APOE* and *APOC1* loci in Alzheimer’s disease. Multi-layered integrative visualization of the *APOE* genomic region (chromosome 19q13.32). Top panel: AD GWAS association signals (−log_10_P) with a prominent peak at the *APOE*-*APOC1*-TOMM40 cluster; the red dot indicates the strongest signal and the horizontal dotted line represents the genome-wide significance threshold (p=5 × 10^−8^). Second panel: brain cortex eQTL associations for *APOC1* from the BrainMeta dataset (n=2,865), showing strong cis-eQTL signals centered at the *APOE* cluster. Third panel: plasma pQTL associations for *APOE* protein levels from the FENLAND study (n=10,708), with extremely strong signals (−log_10_p>100) reflecting robust genetic regulation of *APOE* protein abundance. Fourth panel: plasma pQTL associations for *APOC1* protein levels, demonstrating similarly intense peaks consistent with shared regulatory architecture within the *APOE* cluster. Bottom panel: ENCODE/Roadmap Epigenomics chromatin state annotations across diverse cell and tissue types; color-coded chromatin states include active TSS (red), active enhancer (orange), transcribed region (green), weak enhancer (yellow), repressed Polycomb (blue), and quiescent regions (gray). Gene track displays annotated genes including *APOE* and *APOC1* with corresponding genomic coordinates.

Additionally, we performed exploratory SMR analysis on the well-established *APOE* locus (chromosome 19q13.32), although *APOE* and *APOC1* are not part of our identified gene set (Figure 6). This analysis revealed highly significant overlap between AD GWAS signals and both pQTL (FENLAND) and brain eQTL (BrainMeta) data for *APOE* and *APOC1*. The top pQTL SNP for *APOE* (rs5117) showed extremely strong association (p_eQTL=4.54 × 10^−109^) with SMR causal p-value reaching 1.24 × 10^−48^. Similarly, *APOC1* pQTL (rs439401) and brain eQTL (rs59325138) demonstrated significant associations (p_SMR=8.99 × 10^−15^ and 4.12 × 10^−09^, respectively). However, all HEIDI tests were highly significant (p_HEIDI<10^−10^), indicating extensive horizontal pleiotropy within this region. These results suggest that while the *APOE* cluster shows strong genetic associations with AD, conditional analyses or functional validation are required to establish definitive causal relationships due to the complex linkage patterns at this locus.

### Molecular docking analysis of 6PPD-Q with core target proteins

To evaluate the potential direct binding interactions between 6PPD-Q and core target proteins, we performed molecular docking analysis on six key targets identified through integrated PPI, SMR, and SHAP analyses.

**Figure 7:**
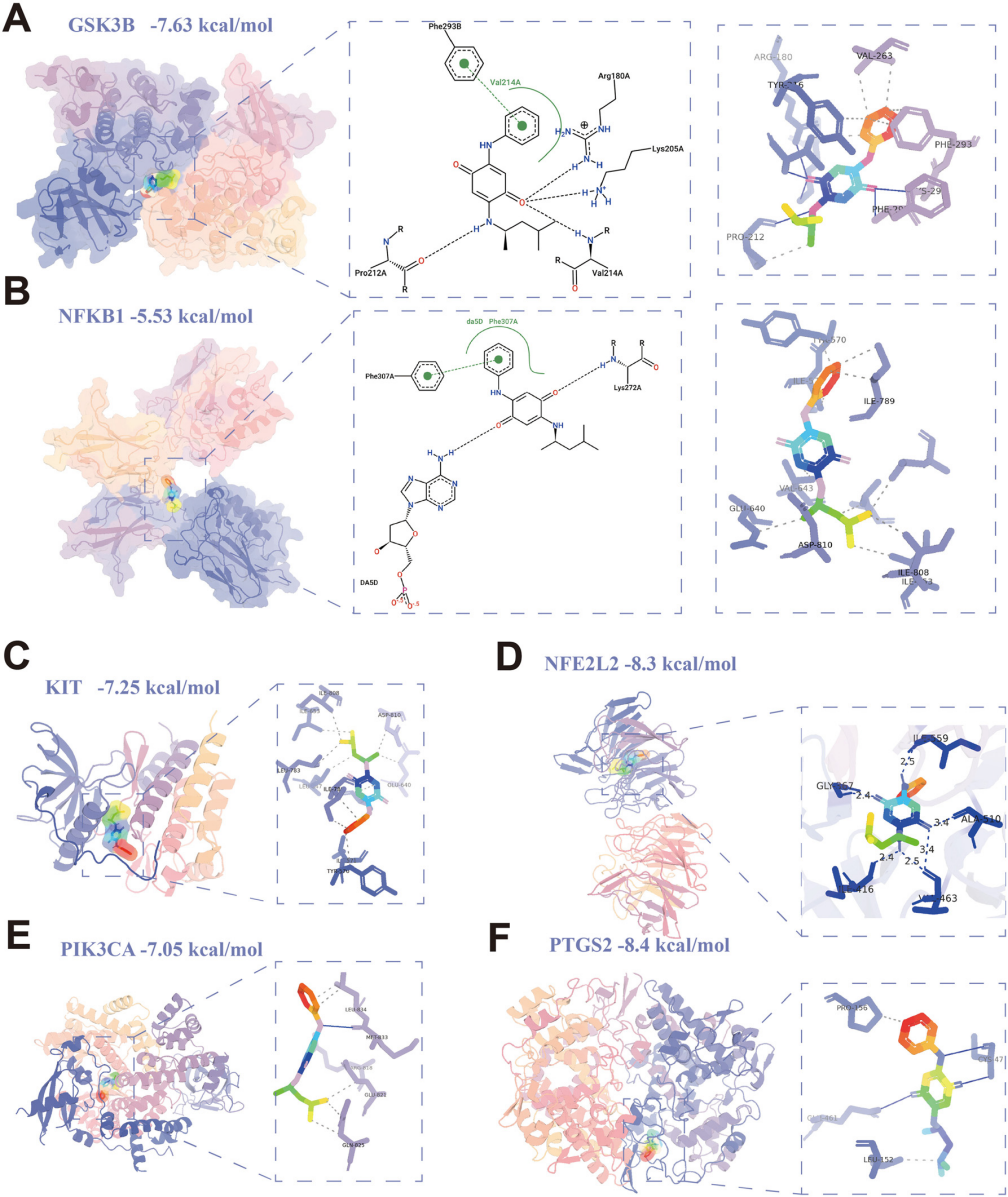
Molecular docking analysis of 6PPD-Q with core target proteins identified through integrated PPI, SMR, and SHAP analyses. (A–F) Predicted binding conformations of 6PPD-Q with six core target proteins: (A) *GSK3B* (−7.63 kcal/mol), (B) *NFKB1* (−5.53 kcal/mol), (C) *KIT* (−7.25 kcal/mol), (D) *NFE2L2* (−8.3 kcal/mol), (E) *PIK3CA* (−7.05 kcal/mol), and (F) *PTGS2* (−8.4 kcal/mol). Left panels display surface binding modes; center and right panels illustrate detailed molecular interactions. Green arcs indicate *π–π* stacking interactions; blue dashed lines indicate hydrophobic contacts; solid lines indicate hydrogen bonds.

Docking simulations revealed variable binding affinities across all tested targets (Figure 7). *PTGS2* exhibited the strongest conventional binding affinity (−8.4 kcal/mol), with 6PPD-Q forming hydrogen bonds with key active site residues and extensive hydrophobic interactions (Figure 7F). *GSK3B* showed strong binding (−7.63 kcal/mol) in the ATP-binding pocket, with interactions including hydrogen bonds and *π–π* stacking (Figure 7A). *KIT* (−7.25 kcal/mol) and *PIK3CA* (−7.05 kcal/mol) demonstrated comparable binding affinities (Figure 7C–E), while *NFKB1* exhibited moderate binding (−5.53 kcal/mol) (Figure 7B). Notably, 6PPD-Q exhibited a binding affinity of −8.3 kcal/mol with *NFE2L2*, within the typical range for non-covalent protein–ligand interactions and comparable to other core targets in this study (Figure 7D).

### *In silico* perturbation analysis reveals disease-amplified transcriptional consequences of key gene knockout in microglia

To evaluate the functional relevance of the identified key targets within the AD transcriptional landscape, we characterized their single-cell expression patterns across the prefrontal cortex snRNA-seq dataset (GSE157827; 163,824 nuclei). Unsupervised clustering and marker-based annotation identified seven major brain cell types (Fig. S1, left), with AD and control nuclei broadly interdigitated across most clusters (Fig. S1, middle). UMAP feature plots demonstrated that *NFKB1*, *GSK3B*, *PIK3CA*, and *NFE2L2* were broadly expressed across multiple cell types, whereas *PTGS2* displayed sparse low-level expression and *KIT* was confined to a discrete subpopulation (Fig. S2).

Perturbation analysis was focused on microglia (n=7,282; 4.4 % of total nuclei). Co-expression network analysis revealed that *NFKB1*, *PIK3CA*, *PTGS2*, and *KIT* each showed exclusively positive correlations with all 50 top-ranked downstream targets, while *GSK3B* and *NFE2L2* each exhibited 88 % positive correlations.

*In silico* knockout simulation revealed distinct perturbation profiles across the six targets (Fig. S3). *NFKB1* knockout produced transcriptional suppression of *B2M, HSPH1, and DIAPH2*. *GSK3B* knockout generated a bidirectional response, with suppression of *LRMDA, SFMBT2*, and *SRGAP2B* alongside de-repression of mitochondrial respiratory chain components, with MT-ND3 and MT-CO3 showing the largest predicted increases (ΔE_AD=+0.20 for both). *NFE2L2* knockout produced a similar mitochondrial de-repression pattern, with MT-CO3 and MT-CO1 showing the strongest positive effects (ΔE_AD=+0.18 and +0.17, respectively). In both cases, the magnitude of predicted de-repression was greater in AD than in control microglia. *PIK3CA* knockout predominantly suppressed *IL13RA1, CAMK2D,* and *ADAM28*. *PTGS2* and *KIT* knockout produced markedly weaker transcriptional effects, with mean absolute effect sizes an order of magnitude below those of the remaining targets.

Comparative analysis of mean absolute knockout effect sizes confirmed a clear hierarchy of transcriptional influence (Fig. S4). *GSK3B* exhibited the highest perturbation effect in both AD (mean |ΔE|=0.113) and control (0.105) conditions, followed by *NFE2L2* (AD: 0.086; Ctrl: 0.084), *PIK3CA*, and *NFKB1*, while *PTGS2* and *KIT* ranked at the bottom. All genes clustered near the diagonal of the AD-versus-control scatter plot, with *GSK3B* and *NFE2L2* showing marginally amplified effects in AD. These findings are consistent with the molecular docking results, in which *NFE2L2* exhibited relatively high predicted binding affinity for 6PPD-Q (SuperPred probability=0.814), and with the SHAP-based feature importance analysis, which ranked both *GSK3B* and *NFE2L2* among the top contributors to AD classification.

## Discussion

Although the role of environmental pollutants in AD pathogenesis has received increasing attention, the potential neurological hazards of the emerging pollutant 6PPD-Q to humans remain insufficiently characterized. Through integrating network pharmacology, transcriptomic validation, and machine learning strategies, this study systematically revealed the molecular associations between 6PPD-Q and AD, identifying key core targets including *NFKB1*, *GSK3B*, *PIK3CA*, *PTGS2*, and *NFE2L2*. Notably, FUMA tissue enrichment analysis demonstrated that these targets exhibited a significant enrichment shift from peripheral digestive and immune organs toward the central nervous system (CNS), particularly the cerebral cortex, basal ganglia, and hippocampus, suggesting that 6PPD-Q may interfere with AD pathological processes through multidimensional molecular networks spanning both peripheral and central systems. Previous studies have confirmed that 6PPD-Q can cross the blood-brain barrier (BBB) to enter the CNS, and human biomonitoring data indicate widespread presence of this pollutant in cerebrospinal fluid (CSF), blood, and urine, likely resulting from translocation from peripheral exposure routes (such as PM2.5 inhalation and dust ingestion) to the central system. Regarding neurotoxic mechanisms, 6PPD-Q can disrupt neurotransmitter synthesis, ion channel function, and GABA receptor activity, while promoting lipid accumulation, enhancing ROS generation, and inducing oxidative damage [42]. Animal experiments have further demonstrated that mice subjected to chronic 6PPD-Q exposure exhibit compromised BBB integrity accompanied by significant cognitive deficits [43]. In neuronal models, 6PPD-Q induces mitochondrial dysfunction and exacerbates α-synuclein aggregation a core pathological hallmark of Parkinson’s disease (PD) and 6PPD-Q levels are significantly elevated in the CSF of PD patients [44]. Given that AD and PD share similar pathological foundations including oxidative stress, protein misfolding, and neuroinflammation, this evidence collectively supports the potential involvement of 6PPD-Q in AD pathological processes, warranting in-depth investigation of its specific molecular targets.

*NFKB1* was identified as the core node with the highest connectivity in the PPI network, and SMR analysis supported a potential causal association between its brain tissue expression level and AD risk. As a pivotal transcription factor in inflammatory responses, sustained activation of the NF-κB pathway is a key factor driving pro-inflammatory polarization of microglia and Aβ deposition. Previous toxicological studies have demonstrated that 6PPD-Q, as a typical quinone compound, possesses the chemical capacity to undergo intracellular redox cycling and generate ROS [45], which serves as an upstream initiating factor for NF-κB signaling activation. Studies in zebrafish models have shown that 6PPD induces lipid metabolism disorders and inflammatory responses through regulating the PPARγ/NF-κB pathway, including elevated levels of pro-inflammatory cytokines TNF-α, IL-1β, and IL-6 [46], a finding also supported by the research of Mengzhu Lv et al. [47]. However, the direction of the causal effect between *NFKB1* and AD requires cautious interpretation. On one hand, in the glial inflammatory microenvironment of AD, excessive NF-κB activation may exacerbate neurodegeneration induced by phosphorylated tau and Aβ deposition. On the other hand, in healthy neurons, NF-κB participates in synaptic plasticity regulation and neurotransmission, and NF-κB dependent DNA double-strand break repair mechanisms may be crucial for learning and memory functions as well as neuronal integrity [48]. Our transcriptomic analysis revealed a declining trend of *NFKB1* expression in AD patient brain tissue, while SMR analysis more likely reflects a negative causal effect at the genetic level, collectively suggesting that NF-*κ*B signaling under basal conditions may exert a protective role against AD.

Meanwhile, this study found that *NFE2L2*, a key regulator of the antioxidant defense system, is also a potential target of 6PPD-Q. Molecular docking results revealed low binding free energy between 6PPD-Q and the *NFE2L2* protein, indicating strong molecular affinity. The *NFE2L2*/ARE pathway is a core signaling cascade for maintaining cellular redox homeostasis. SHAP analysis indicated that *NFE2L2* exerts a protective effect against AD, and transcriptomic data also showed that its expression levels in young individuals were significantly higher than those in elderly populations and AD patients. In human hepatocyte and lung cell models, 6PPD-Q exposure induced ROS production in a dose-dependent manner and upregulated *NFE2L2* mRNA expression [49], 50], suggesting that activation of the Nrf2 pathway may represent an adaptive compensatory response to 6PPD-Q-induced oxidative damage. *In silico* knockout of *NFE2L2* in microglia predicted de-repression of mitochondrial respiratory chain components MT-CO3 and MT-CO1 (ΔE_AD=+0.18 and +0.17, respectively), with greater effect magnitudes observed in AD than in control nuclei, providing cell-type-resolved transcriptional evidence for its regulatory role in mitochondrial homeostasis under AD conditions.

Dysregulation of intraneuronal kinase signaling networks represents another important pathological feature of AD. *GSK3B* and *PIK3CA* identified in this study may be involved in this pathological process. *GSK3B* is a core kinase driving tau protein hyperphosphorylation and neurofibrillary tangle formation. Human Protein Atlas data indicate that *GSK3B* is highly enriched in cerebral cortex neurons, displaying strong cytoplasmic/membrane staining signals, while showing relatively lower expression in glial cells and endothelial cells. Molecular docking results demonstrated that 6PPD-Q exhibits strong binding affinity with both *GSK3B* and *PIK3CA*, suggesting that 6PPD-Q may directly interfere with the functional activity of these two key kinases. The PI3K/AKT pathway is highly enriched in the CNS and is essential for maintaining synaptic plasticity and learning and memory functions in rodents. Recent studies have shown that oral exposure to 6PPD-Q in mice can inhibit the PI3K/AKT pathway, subsequently inducing metabolic syndrome and cognitive deficits [51]. Another study further revealed that gut microbiota can mediate 6PPD-Q-induced cognitive impairment through inhibition of the PI3K/AKT signaling pathway [52]. This evidence collectively suggests that 6PPD-Q may exert neurotoxic effects through binding to *GSK3B* and *PIK3CA*. *PTGS2* (COX-2) demonstrated certain diagnostic predictive value in our machine learning model. As a classic inflammatory marker, *PTGS2* has been reported in various environmental exposures and inflammatory diseases [45], 53]. Although its specificity is relatively limited, it may still serve as a potential target reflecting 6PPD-Q-induced inflammatory responses.

The molecular-level damage described above may ultimately converge on synaptic function deterioration. GO functional enrichment analysis in this study revealed that common targets between 6PPD-Q and AD were enriched in structural components such as synaptic membrane and postsynaptic density, and the loss of synaptic plasticity is one of the important neurobiological bases for cognitive decline in AD. Taken together, 6PPD-Q may synergistically promote synaptic structural damage and neurotransmission dysfunction through multiple mechanisms including oxidative stress induction, neuroinflammation, and kinase signaling dysregulation.

This study provides a theoretical framework for the neurotoxicity of 6PPD-Q; however, several limitations should be acknowledged. Network pharmacology and molecular docking are primarily based on computational predictions, and the predicted binding affinity between 6PPD-Q and core targets such as *NFKB1*, while suggestive, does not necessarily translate to transcriptional upregulation or functional activation under physiological conditions. Experimental validation is therefore required to demonstrate the specific regulatory effects of 6PPD-Q on these targets at the molecular and cellular levels. Additionally, the transcriptomic datasets employed in this study were derived from post-mortem brain tissues of late-stage AD patients and were used solely to evaluate whether the computationally identified 6PPD-Q targets exhibit disease-relevant expression alterations in AD brain tissue; they do not reflect individual 6PPD-Q exposure levels, and no direct inference regarding exposure-response relationships should be drawn from these data. Late-stage pathological alterations may furthermore obscure the primary molecular events associated with early environmental exposure. The sample size of GSE159699 was relatively limited (AD group n=12), and although cross-validation using GSE174367 (n=90) yielded consistent directional expression patterns for core targets, larger cohorts are warranted for further validation. Future research should focus on establishing chronic low-dose exposure animal models to verify whether 6PPD-Q can penetrate the BBB and induce pathological alterations in these core targets, thereby providing more direct biological evidence for the role of environmental factors in AD pathogenesis.

## Conclusions

This study provides the first systematic investigation of the potential molecular associations between 6PPD-Q exposure and AD pathogenesis. By integrating network pharmacology, transcriptomic validation, machine learning, and Mendelian randomization analysis, we identified key targets including *NFKB1*, *GSK3B*, *PIK3CA*, *NFE2L2*, and *PTGS2* that may mediate 6PPD-Q neurotoxicity. Our findings suggest that 6PPD-Q may participate in AD-related pathological processes through multiple mechanisms, including oxidative stress induction, neuroinflammatory cascade activation, and kinase signaling network disruption, ultimately converging on synaptic dysfunction.

## Supplementary Material

Supplementary Material

Supplementary Material

Supplementary Material

Supplementary Material

Supplementary Material

Supplementary Material

Supplementary Material
