# Supplementary material for: 6PPD-quinone exposure and Alzheimer’s disease: insights from integrative network pharmacology, transcriptomics, machine learning, and molecular docking
Source: Open Med (Wars). 2026 Jun 24;21(1):20261477. doi: 10.1515/med-2026-1477 (PMC13290095; doi:10.1515/med-2026-1477)
Supplement: Supplementary file 6 — Supplementary Material [file j_med-2026-1477_suppl_006.docx]

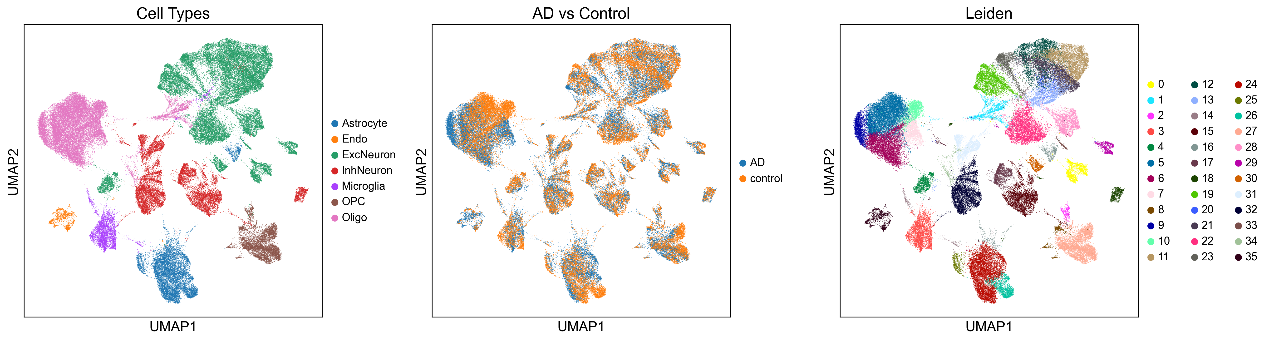


**Fig. S1. UMAP visualization of the GSE157827 prefrontal cortex snRNA-seq dataset.** (Left) Cell type annotation of 163,824 nuclei across seven major brain cell types: excitatory neurons (ExcNeuron), inhibitory neurons (InhNeuron), oligodendrocytes (Oligo), oligodendrocyte precursor cells (OPC), astrocytes, endothelial cells (Endo), and microglia. (Middle) Distribution of AD and control nuclei across the UMAP embedding. (Right) Unsupervised Leiden clustering (resolution = 0.5) identifying 36 transcriptionally distinct clusters.


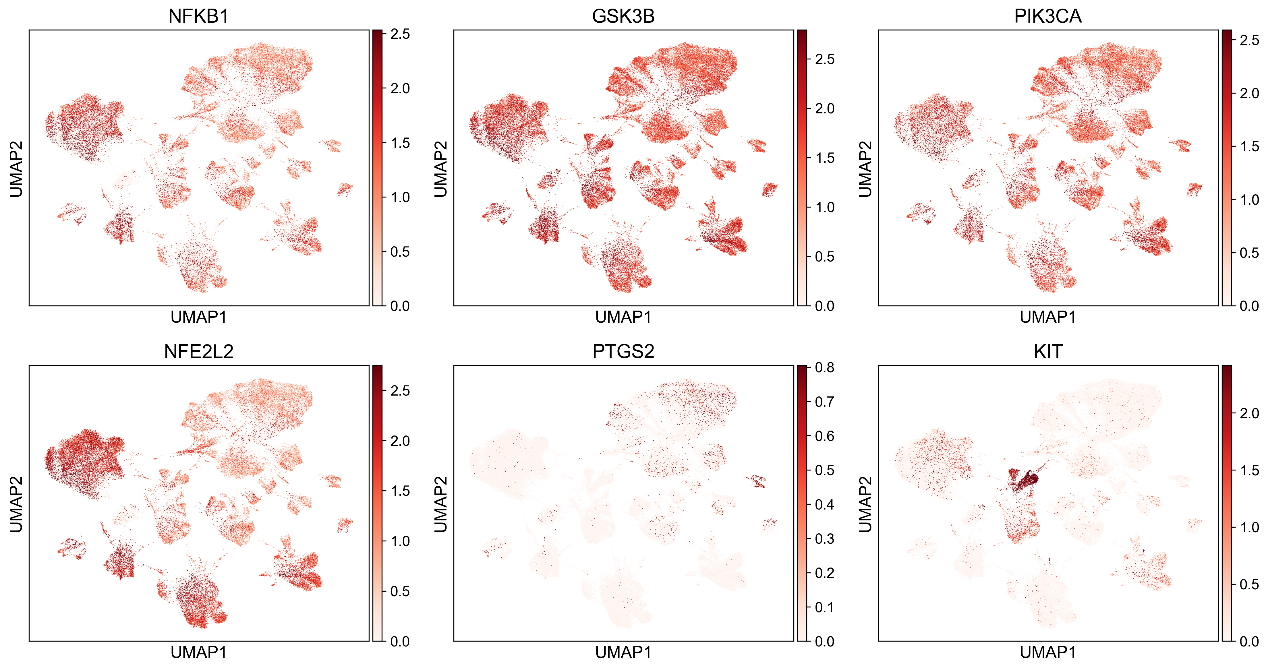


**Fig. S2. UMAP feature plots of six key target genes in the prefrontal cortex.** Log-normalized expression of NFKB1, GSK3B, PIK3CA, NFE2L2, PTGS2, and KIT overlaid on the UMAP embedding. Color intensity (white to deep red) reflects expression level on a per-gene scale. NFKB1, GSK3B, PIK3CA, and NFE2L2 display broad multi-cell-type expression, whereas PTGS2 exhibits sparse low-level expression and KIT is restricted to a discrete subpopulation.


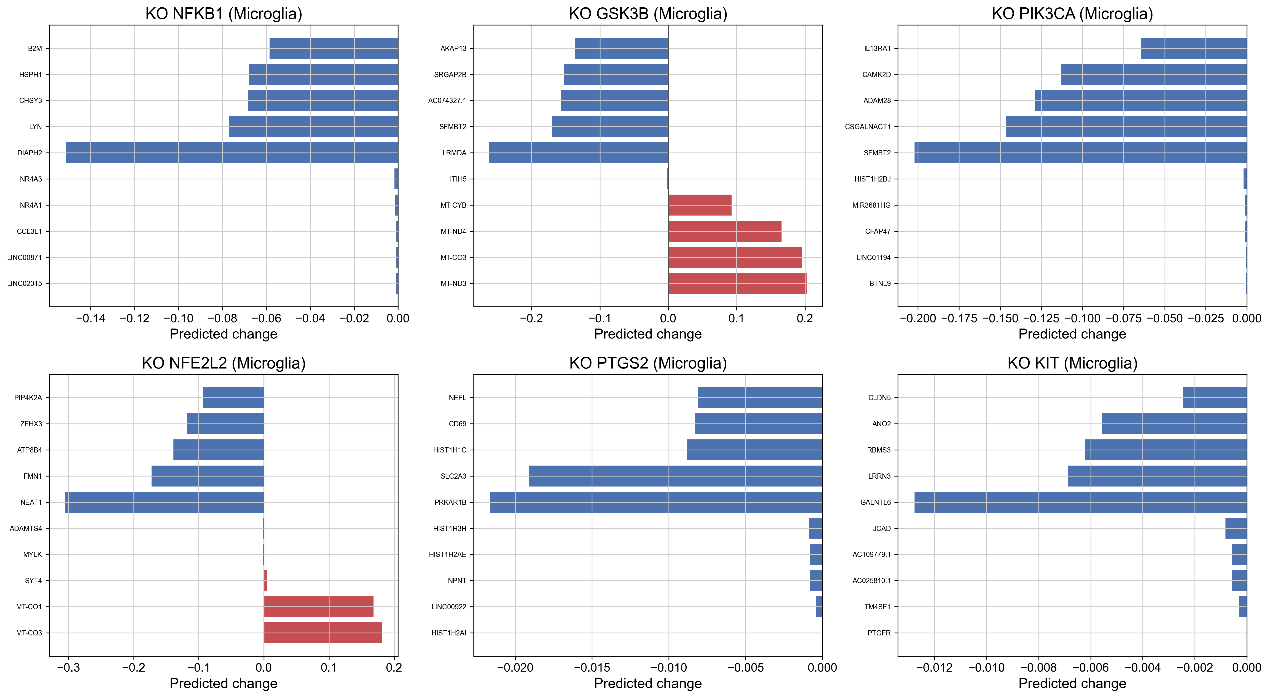


**Fig. S3. Predicted downstream transcriptional effects of in silico gene knockout in microglia.** Horizontal bar plots showing the top predicted target genes (5 most suppressed and 5 most de-repressed) for each of the six key gene knockouts (NFKB1, GSK3B, PIK3CA, NFE2L2, PTGS2, KIT) in microglial nuclei. Blue bars indicate predicted transcriptional suppression (negative ΔE); red bars indicate predicted de-repression (positive ΔE). Values represent predicted effect sizes in AD microglia (ΔE_AD = −r × E_target).


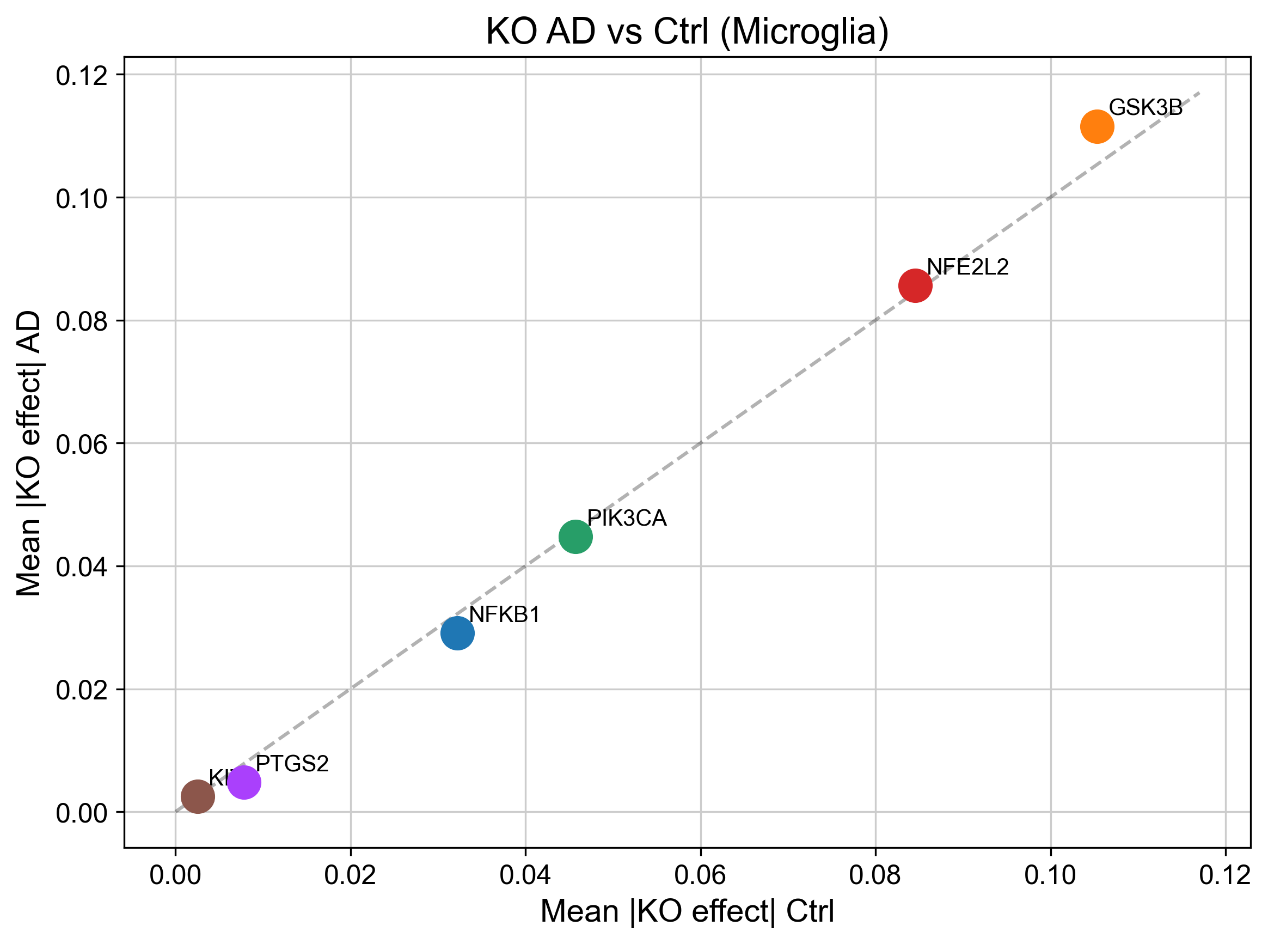


**Fig. S4. Comparison of mean absolute knockout effect sizes between AD and control microglia.** Scatter plot of mean absolute predicted knockout effect size in AD (y-axis) versus control (x-axis) microglia for each key gene. The dashed diagonal line represents equal effect in both conditions. Points above the diagonal indicate greater AD-specific transcriptional impact.
