# Supplementary material for: 6PPD-quinone exposure and Alzheimer’s disease: insights from integrative network pharmacology, transcriptomics, machine learning, and molecular docking
Source: Open Med (Wars). 2026 Jun 24;21(1):20261477. doi: 10.1515/med-2026-1477 (PMC13290095; doi:10.1515/med-2026-1477)
Supplement: Supplementary file 7 — Supplementary Material [file j_med-2026-1477_suppl_007.docx]

**Supplementary Materials**

Supplementary Data 1. 6PPD-Q Targets.

Supplementary Data 2. Alzheimer's disease genes.

Supplementary Data 3. GO and KEGG analysis results.

Supplementary Data 4. PPI analysis results.

Supplementary Data 5. SMR analysis results.

Supplementary Figure S1. UMAP visualization of cell type annotation, disease condition, and Leiden clustering in the GSE157827 prefrontal cortex snRNA-seq dataset.

Supplementary Figure S2. UMAP feature plots of six key target genes across the prefrontal cortex.

Supplementary Figure S3. Predicted downstream transcriptional effects of in silico gene knockout in microglia.

Supplementary Figure S4. Comparison of mean absolute knockout effect sizes between AD and control microglia.
